# Biochar Amendment Stimulates Utilization of Plant-Derived Carbon by Soil Bacteria in an Intercropping System

**DOI:** 10.3389/fmicb.2019.01361

**Published:** 2019-06-18

**Authors:** Hongkai Liao, Yaying Li, Huaiying Yao

**Affiliations:** ^1^Key Laboratory of Urban Environment and Health, Institute of Urban Environment, Chinese Academy of Sciences, Xiamen, China; ^2^Key Laboratory of Urban Environmental Processes and Pollution Control, Ningbo Urban Environment Observation and Research Station-NUEORS, Chinese Academy of Sciences, Ningbo, China; ^3^College of Advanced Agricultural Sciences, University of Chinese Academy of Sciences, Beijing, China; ^4^Guizhou Provincial Key Laboratory of Mountain Environment, Guizhou Normal University, Guiyang, China; ^5^Research Center for Environmental Ecology and Engineering, School of Environmental Ecology and Biological Engineering, Wuhan Institute of Technology, Wuhan, China

**Keywords:** biochar, plant-derived carbon, ^13^CO_2_ steady-state labeling, stable-isotope probing, intercropping, rhizosphere microbes

## Abstract

Plant-derived carbon (C) is considered fundamental to understand the interaction between rhizosphere microbes and plants in terrestrial ecosystems. Biochar soil amendment may enhance plant performance via changing soil properties or microbial diversity in the rhizosphere. However, our knowledge of how plant-microbiome associations respond to biochar amendment remains rather limited. Herein, ^13^CO_2_ steady-state labeling combined with DNA stable-isotope probing was used to characterize soil bacterial communities in the rhizosphere contributing to the utilization of plant-derived C. The diversity of bacteria active in the utilization of root exudates was determined after biochar amendment in a legume-based intercropping system (*Vicia faba* L., with *Zea mays* L.). The results showed the biochar application not only changed the bacterial community structure and diversity in the rhizosphere, but also altered bacterial members actively assimilating plant-derived C. There were more labeled species in the biochar-amended soils than the control soils. Compared with the control, the biochar amendment increased the relative abundances of Firmicutes and Bacteroidetes members (i.e., *Bacillus*, *Clostridium*, *Sporomusa*, *Desulfosporosinus*, and *Alicyclobacillus*) while decreasing the abundances of Proteobacteria members (e.g., *Methylobacterium* and *Sphingomonas*) utilizing plant-derived C. In contrast, slow-growing species of the phyla Acidobacteria, Planctomycetes, and Gemmatimonadetes were barely labeled. The bacteria found stimulated by the biochar amendment are known for their ability to fix nitrogen, solubilize phosphorus, or reduce iron and sulfur, which may potentially contribute to the “biochar effect” in the rhizosphere. This study is the first to provide empirical evidence that biochar amendment can alter the soil bacterial community assimilating plant-derived C; this may have consequences for nutrient cycling and improving plant performance in intercropping systems.

## Introduction

Modern agricultural systems provide high crop yields, but they also generate serious impacts on the environmental. For example, intensive use of mineral fertilizers and pesticides leads to such soil acidification (Gollany et al., 2005), groundwater contamination (Gollany et al., 2004), and increasing greenhouse gas emissions (Dalal et al., 2003). Intercropping is an ancient and traditional agricultural practice, and especially legume-based intercropping systems have great potential for contributing to agricultural productivity through increasing nitrogen (N) inputs and phosphorous (P) bioavailability (Rose et al., 2015; Tang et al., 2016) in the terrestrial system. In this regard, an intercropping system provides a way to introduce soil available nutrients into the agro-ecosystem, thus avoiding the excessive use of fertilizers and pesticides relied upon during conventional cultivation. Furthermore, a recent meta-analysis indicated that intercropping can increase crop yields in Africa by an average of 23% compared with monocropping (Himmelstein et al., 2017). Hence, an improved understanding of nutrient cycling in intercropping systems may offer new insights into achieving long-term agricultural productivity.

Plants and microbiomes build and engage in a complex and varied molecular “dialogue” in the rhizosphere (Gkarmiri et al., 2017). It has been estimated that rhizodeposition accounts for 17% of total photoassimilated carbon (C), and belowground C allocation by grasses was higher than crops (Nguyen, 2003; Pausch and Kuzyakov, 2018). The beneficial effects of an intercropping system are closely related to the partitioning of C that occurs belowground and its utilization by different functional groups of microbes (Fan et al., 2008). However, this soil microbial community is often influenced by plant species due to differences in the quantity and quality of C resources produced by root exudates (Garland, 1996; Liu et al., 2017). Additional factors controlling plant C allocation belowground include drought events, water status, and fertilization regimes (Drigo et al., 2010; Yao et al., 2012; Fuchslueger et al., 2016; Wang et al., 2016). Nevertheless, plants can actively select beneficial microbes or disrupt the invasion of pathogenic microbes in the rhizosphere by secreting particular root exudates (Chapelle et al., 2016). Meanwhile, shifts in the microbial community consuming plant-derived C may have strong effects on plant development and soil nutrient cycling (Hannula et al., 2017).

Biochar is a C-rich product of biomass pyrolysis intended for use as a soil amendment. Biochar amendment has been found to increase both the soil water-holding capacity (Abel et al., 2013) and nutrient availability (Zheng et al., 2018), and to also increase the pH in acidic soil (Whitman et al., 2016). Some studies have shown that biochar-induced changes in soil properties (e.g., pH and nutrient availability) may have beneficial effects on crop productivity (Lehmann et al., 2011; Liu et al., 2017). However, it is worth noting that application of the biochar into soil would be expected to have wide-ranging effects, depending on the quality of the biochar in terms of feedstock and pyrolysis temperature (Jaiswal et al., 2014; Imparato et al., 2016). Recently, some studies have shown that biochar amendment may increase the diversity of soil microbes; this phenomenon, may be contributed to the release of non-labile biochar-associated organic compounds or the changes in soil properties induced by biochar amendment (Kolton et al., 2011; Lehmann et al., 2011; Zhang et al., 2017). Kolton et al. (2017) found that biochar-enhanced plant performance was related not only to greater microbial richness but also linked to higher metabolic potential in the rhizosphere. However, it is still an open question whether biochar applications can facilitate soil microbes assimilating plant-derived C, leading to a tight-knit plant-microbiome association in the rhizosphere. Moreover, numerous studies tend to focus on the overall microbial community and its diversity in the rhizosphere of monocropping systems. By contrast, how biochar amendment may affect soil microbial community composition and diversity in intercropping systems is understudied. Plants can release a variety of root exudates into the rhizosphere, and these biologically active compounds from root exudate are known to influence the rhizosphere microbiome (Baetz and Martinoia, 2014; Mendes et al., 2017). Salicylic acid and γ-aminobutyric acid concentrations have been shown to be correlated with specific taxa that are enriched in the rhizosphere (Badri et al., 2013; Zhalnina et al., 2017). Biochar amendment may change the influences of root exudates on the rhizosphere microbiome. For example, biochar may alter rhizosphere microbial communities, facilitating propagation of microbes via absorption of root exudates (Gu et al., 2017) or via physical attributes generated by the porous properties of biochar (Pietikäinen et al., 2000).

In the present study, we performed ^13^CO_2_ steady-state labeling coupled with DNA-stable isotope probing (SIP) and investigated soil bacterial diversity via high-throughput amplicon sequencing in an intercropping system. The objectives of the present study were: (1) to explore the effects of biochar amendment on soil bacterial communities actively utilizing plant-derived C, and (2) to understand the interactions between root exudates and soil bacterial communities after biochar amendment in an intercropping system. Our hypothesis was that biochar amendment not only stimulates bacterial species affiliated with the phyla Bacteroidetes and Firmicutes, but also alters a subset of bacterial communities consuming plant-derived C in the rhizosphere, leading to a distinct plant-microbe association in the intercropping system.

## Materials and Methods

### Biochar Preparation and Soil Sampling

The biochar used in this experiment was made from apple (*Malus pumila* Mill.) wood chip, a common agricultural solid waste in China. Air-dried apple wood chips were pyrolyzed at 500°C for 5 h in a closed container under oxygen-limited condition, with nitrogen gas as the medium gas in a muffled furnace (Isotemp, Fisher Scientific, United States). More details of biochar preparation are provided by Xu et al. (2014). The biochar had a pH value of 10.9 (1:2.5 water); the C content was 73.6%; and the N content was 0.6%. The ratio of mass loss upon biochar production was 77%.

In August 2016, soil was collected from a depth of 0–5 cm in a fully managed field in Ningbo (29°56′21″N, 121°35′23″E), Zhejiang Province, China. The soil is a Plinthosol (FAO/UNESCO) with clay loam texture. The soil pH, total C, and total N were 6.89 (1:2.5 water), 15.3 g kg^–1^, and 2.1 g kg^–1^, respectively. After removing plant residues and stones, the fresh soils were passed through a 2-mm sieve and stored in a 300-L plastic bucket at the room temperature for 7 days until use.

### Experimental Design

In order to explore the effect of biochar amendment on bacterial communities in the rhizosphere, a relatively high ratio of biochar amendment (2%, w/w) was selected. This biochar rate was also used in a field experiment in an intercropping system (Liu et al., 2017). Soil was amended with biochar and 250 g amount of soil (dry weight basis) was added to each plastic pot (5.3 cm diameter, 13.2 cm height). Soil water-holding capacities of control and biochar treatments were measured by gravimetrically following saturation and free-draining, and then different mass of water was added in control and biochar treatment separately to reach approximately 40% water-holding capacity. A legume plant, faba bean (*Vicia faba* L.), and a cereal crop, maize (*Zea mays* L.), were selected to represent the intercropping system. All the seeds were immersed in a 3% H_2_O_2_ solution for 10 min and washed with distilled water, then germinated in Petri dishes covered with wet filter papers for 3 days at 28°C in the dark before sowing. One faba bean and one maize seedling were planted in horizontal orientation per pot. These seedling pairs were cultivated in a growth chamber under 14-h light photoperiod at 28°C (day) and 20°C (night) temperatures.

After 2 weeks of growth (i.e., at day 14), a continuous steady-state labeling approach was implemented according to Wang et al. (2016). Briefly, ^13^CO_2_ at 99.8 atom % excess (Sigma-Aldrich, United States) and at ambient CO_2_ concentrations was introduced, with a total ^13^CO_2_ and ^12^CO_2_ concentration of 350 ppm. To achieve this CO_2_ concentration, the flow rates of CO_2_-free air and ^13^CO_2_ into the plant growth chamber (Zhicheng Technology Co., Ltd., Ningbo, China) were set to 9.0 L min^–1^ and 3.15 mL min^–1^, respectively. For the unlabeled chamber, which served as the control, the same gas flow was used except that ^12^CO_2_ was used instead of ^13^CO_2__._ There were four treatments in the experiment (unlabeled-non-biochar, unlabeled-biochar, ^13^CO_2_-labeled-biochar, and ^13^CO_2_- labeled-biochar), with three replicates per treatment. Three pots for each treatment were randomly placed into the unlabeled and ^13^CO_2_ labeled chambers, respectively. During this labeling, plants were kept in the growth chamber under the same light and temperature conditions as they had before. Soil water conditions were maintained by adding deionized water every 2 days by weight for both control and biochar treatments during the 35-day labeling period.

**FIGURE 1:**
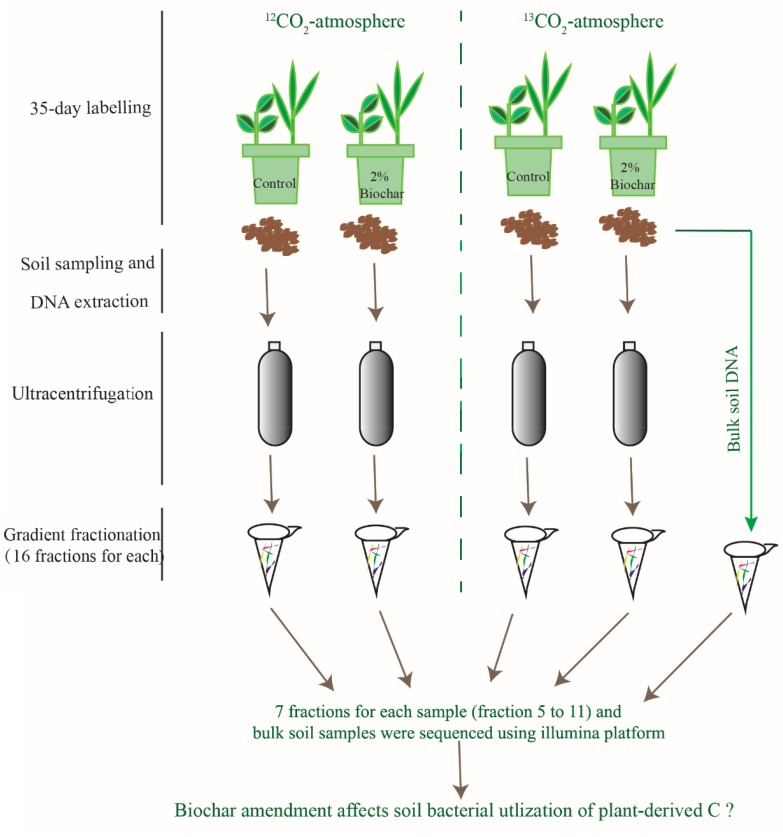
A conceptual diagram for the study design, methods and objective.

The unlabeled and ^13^CO_2_-labeled plants and soils were destructively sampled at day 49 (i.e., after 35-day of continuous labeling). The image of plants in the pots is shown in the Supplementary Figure S1. The roots and shoots were washed with water, then dried for 45 min at 105°C and again at 65°C for 48 h to measure their biomass. Since the pot was small in size the root growth in each pot was extremely extensive, all soil in a given pot was considered as rhizosphere soil. Soil samples from each pot were immediately freeze-dried for later DNA extraction. An overall conceptual diagram for the study design is shown in Figure 1.

### DNA Extraction

Total genomic DNA was extracted from 0.50 g of freeze-dried rhizosphere soil samples using the Fast DNA SPIN Kit for soil (MP Biomedicals, Santa Ana, CA, United States) following the manufacturer’s protocols. The DNA concentration was measured using a NanoDrop^TM^ 2000 spectrophotometer (Thermo Scientific, Waltham, MA, United States).

### Enrichment Analysis Using ^13^C

Two micrograms of DNA was placed into a tin capsule (Thermo Scientific), and dried for approximately 2.5 h at 50°C. Freeze-dried rhizosphere soil samples from the unlabeled and ^13^CO_2_-labeled chambers were milled to pass through a 200-mesh sieve. Between 2 and 4 mg of soil from each sample was weighed, and both the DNA and soil samples were examined with an elemental analyzer (Thermo Scientific FLASH 2000, Germany) coupled online to an isotope ratio mass spectrometer (Delta V advantage, Thermo Finnigan, Germany). The δ^13^C (‰) was calculated as δ^13^C (‰) = [(Rsample – Rstandard)/Rstandard] × 1000, where R is the molar ratio of ^13^C to ^12^C. DNA concentration in the ^13^C-labeled sample was calculated as ^13^C-DNA (ng g^–1^ soil) = ^13^C atom% excess × DNA concentration (ng g^–1^ soil), where ^13^C atom% excess = ^13^C atom% of the samples from ^13^CO_2_-labeled chamber –unlabeled chamber.

### Soil Physicochemical Analysis

The pH was measured in a soil-to-water ratio of 1:2.5 (w/v) with a pH-meter (Bao, 1999). Soil organic C and total N were measured with an element analyzer (vario MACRO cube, Elemental, Germany). Soil Olsen-P was extracted with 0.5 M of NaHCO_3_ in a soil-to-water ratio of 1:10 (w/v) for 30 min and measured following the methods described by Olsen (1954). Soil nitrate (NO_3_^–^) and ammonium (NH_4_^+^) were extracted with 2 M of KCl in a soil-to-water ratio of 1:10 (w/v) for 30 min, and then determined by using an automated continuous flow analyzer (AutoAnalyzer3, SEAL, Germany).

### Caesium Chloride Ultracentrifugation

From each sample, 3 μg of DNA was fractionated by caesium chloride (CsCl) equilibrium density gradient ultracentrifugation, as described by Long et al. (2018). Briefly, the CsCl solution was added to a 5.1-mL Quick-Seal polyallomer centrifuge tube (Beckman Coulter, Brea, CA, United States) that contained 1.6914 g mL^–1^ of CsCl verified by an AR200 refractometer (Reichert, Depew, NY, United States). Centrifugation was performed at 180,300 rcf for 44 h at 20°C using a Vti 65.2 vertical rotor (Beckman Coulter). After completing this centrifugation, DNA was collected from the tube bottom to its top in 16 equal fractions (300 μL per fraction) by replacing the gradient medium with sterile water using a single-channel syringe pump. The density of each fraction was then measured using an AR200 refractometer (Reichert). DNA in each fraction was purified with PEG 6000 and 70% ethanol, and then dissolved in 30 μL of double-distilled H_2_O for the downstream analysis.

### PCR Amplification and Illumina Sequencing

Seven DNA fractions for each sample with a buoyant density of 1.67–1.72 g mL^–1^ were analyzed. These DNA fractions were selected because they encompassed most of the extracted DNA in both labeled and unlabeled samples. In addition, six non-fractionated DNA samples (three control and three biochar samples for each) from the ^13^CO_2_-labeled chamber were analyzed. Sequencing of the 16S rRNA gene was done via multiplexed barcoded amplicon sequencing. Primers 515f/907r targeting approximately 392 bp in the V4–V5 hypervariable region of the bacterial 16S rRNA gene were used for PCR amplification (Zhou et al., 2011). Both forward and reverse primers were barcoded with a unique error-correcting eight-base barcode (Hamady et al., 2008). The PCR reaction contained 10 μL of 5 × GoTaq^®^ Green Master Mix (Promega, Fitchburg, WI, United States), 10 μM of each primer, 1 μL of template DNA, and ultraclean water to a volume of 50 μL (done three times). Each sample was amplified in triplicate with the following conditions: 30 cycles of denaturation at 94°C for 30 s, annealing at 55°C for 30 s and extension at 72°C for 30 s, with a final extension at 72°C for 10 min. The triplicate amplicons were pooled and purified using a Universal DNA Purification Kit (Tiangen, Beijing, China). Purified PCR products were quantified with a NanoDrop^TM^ 2000 spectrophotometer (Thermo Scientific), and then sent to Novogene (Beijing, China) for sequencing, Paired-end sequencing (2 × 250 bp) was performed on an Illumina HiSeq2500 platform (Illumina, San Diego, CA, United States).

### Sequence Processing and Analysis

Bacterial sequences were processed using QIIME v1.9.1 (Caporaso et al., 2010). Briefly, forward and reverse reads were merged using FLASH (Fast Length Adjustment of SHort reads) (Magoč and Salzberg, 2011) before downstream processing. Reads with a low average quality score (<20) and short length (<100 nt) were removed (Kong, 2011). Sequences were checked for any chimeras and filtered by the UCHIME algorithm in the USEARCH tool (Edgar, 2010) using the rRNA Gold fasta file of chimer-checked reference sequences. High-quality sequences were assigned to operational taxonomic units (OTUs) with a similarity threshold of 97%, through an open reference OTU-picking strategy using UCLUST. The most abundant sequence in the cluster for each OTU unit was selected as a representative sequence for that OTU, and it was then assigned to taxonomy against the latest version of the Greengenes database (v13_8) (McDonald et al., 2012). Any OTUs affiliated to “chloroplast,” “Archaea,” “mitochondria,” or “unassigned” were discarded from the final OTU table. Quality control removed 1,15,3,09,1 reads, leaving 9,50,0,10,1 high-quality sequences. Individual samples contained from 37,4,06 to 189,218 total sequences each, with a mean number of 105,557 ± 37,159. The 16S rRNA gene sequencing data have been deposited at the National Center for Biotechnology Information short Reads Archive under study PRJNA528377.

### Statistical Analysis

In addition to stable isotope incorporation, buoyant density of a macromolecule is affected by various factors such as the G+C content of DNA (Youngblut and Buckley, 2014). ^13^C-DNA may have the same buoyant density as ^12^C-DNA in the same centrifuge tube (Pepe-Ranney et al., 2016). Hence, it is important to use the unlabeled substrates to gain reliable isotopic labeling results for robust analysis and inference. By comparing heavy gradient fractions in the labeled samples to their corresponding fractions in the unlabeled samples, those active microbes with labeled DNA can be obtained. Here, we applied the DESeq2 package, an RNA-Seq differential expression statistical framework (Love et al., 2014), to gain insight into the bacterial OTUs enriched in the heavy fractions of ^13^C-labeled gradients vs. unlabeled samples. The DESeq2 package contains several features for establishing a statistically robust framework that can account for reading differences in the sequence libraries (Angel et al., 2018). We divided the CsCl gradient fractions into “light” or “heavy” fractions. Values > 1.70 g mL^–1^ were defined as the cut-off concentration for heavy category fractions, since we were mainly interested in OTU enrichment in the heavy fractions. OTUs were filtered independently prior to conducting multiple comparisons. To ensure sufficient data was available for comparisons, a sparsity threshold of 0.35 was selected for the heavy fractions of both unlabeled and ^13^C-labeled gradients (Love et al., 2014). In this way, an OTU not found in at least 35% of the heavy fractions (^13^C-labeled and unlabeled gradients) was removed as statistically uninformative. Removing those OTUs lacking sufficient sequence counts to provide statistically significant results can reduce the multiple redundant comparisons performed (Pepe-Ranney et al., 2016). In addition, a false discovery rate of 0.10 was applied, with the Benjamini-Hochberg method (Benjamini and Hochberg, 1995) applied for the *P*-value corrections. OTUs statistically enriched in the heavy fractions from ^13^C-labeled samples to corresponding unlabeled samples were interpreted as being the labeled bacteria (Pepe-Ranney et al., 2016).

Statistical tests were performed using SPSS 16.0 (SPSS Inc., Chicago, IL, United States). The differences in soil properties, bacterial community composition, and plant biomass between treatments were determined by one-way analysis of variance (ANOVA) with Fisher’s least significant difference (*LSD*) test, and *P* < 0.05 was considered statistically significant. For the 16S rRNA sequencing data, non-metric-multidimensional scaling (NMDS) and principal coordinate ordinations (PCoA) based on Bray-Curtis dissimilarities were applied in R (R Core Team, 2013) with the “phyloseq” package (McMurdie and Holmes, 2013); the results were visualized with the “ggplot2” package (Wickham, 2010). The differences in bacterial community composition between unlabeled and ^13^CO_2_-labeled samples in various fractions were tested using Adonis tests with 999 permutations in the “vegan” package (Oksanen et al., 2016). Additionally, species richness and its associated error were estimated for each sample using the “breakaway” package in R (Willis et al., 2017).

## Results

### ^13^C Enrichment and Soil Properties

**FIGURE 2:**
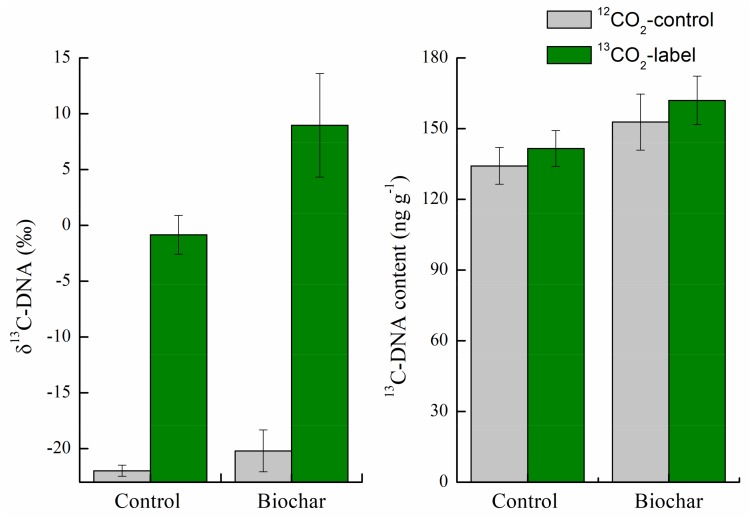
Isotopic signatures of δ^13^C (‰) and ^13^C-DNA concentration (ng g^–1^) in the control and biochar treatments after 35-day of continuous labeling. Bars are means ± SE. Significant differences between treatments were tested by one-way ANOVA with Fisher’s *LSD* test.

After 35-day of continuous labeling, the overall isotopic signatures of δ^13^C were −20.1‰ ± 1.87‰ and 398‰ ± 32.2‰ in the unlabeled and ^13^CO_2_-labeled soils of the control treatment. For the biochar treatment, the corresponding values were −21.9‰ ± 0.49‰ and 335‰ ± 15.8‰ (Supplementary Figure S2). Additionally, relatively high δ^13^C (8.97‰) in the DNA and^13^C-DNA content (162 ng g^–1^) were found in the biochar treatment; the corresponding values were −0.84‰ and 141 ng g^–1^ in the control treatment (Figure 2). These results indicated the successful incorporation of ^13^C-labeled rhizodeposits into soil microbes.

Compared with the control treatment, significantly higher (*P* < 0.05) C/N ratio was found in the biochar amendment soils (Table 1). In addition, relatively high soil pH, Olsen-P, NH_4_^+^, and NO_3_^–^ contents were detected in the biochar treatment, although none of these values were significantly different compared with the control treatment. Similarly, there were no significant differences (*P* > 0.05) in total plant biomass between the biochar (4.65 g dry biomass) and control (4.05 g dry biomass).

**TABLE 1:** Effect of the biochar amendment on soil properties in a legume-based intercropping system.

**Treatment**	**pH (1:2.5 H_2_O)**	**SOC (g kg^–1^)**	**TN (g kg^–1^)**	**C/N**	**Olsen-P (mg kg^–1^)**	**NH_4_+-N (mg kg^–1^)**	**NO_3__–_-N (mg kg^–1^)**
Control	5.35 (0.05)	19.3 (0.26)b	1.97 (0.05)	9.82 (0.16)b	42.2 (1.1)	4.49 (0.10)	0.38 (0.08)
Biochar	5.48 (0.03)	24.8 (0.73)a	2.11 (0.03)	11.8 (0.39)a	44.6 (0.53)	4.74 (0.13)	1.10 (0.35)

*SOC, soil organic carbon; TN, total nitrogen; C/N, carbon to nitrogen ratio; Olsen-P, available phosphorous; NH_4_^+^-N, ammonium nitrogen; and NO_3__–_-N, nitrate nitrogen. Values are the means of the three replicates ± SE; Volumes within a column followed by the same lower case letters are a significant difference at *P* < 0.05 level.*

### Overall Bacterial Community Structure in the Rhizosphere

**FIGURE 3:**
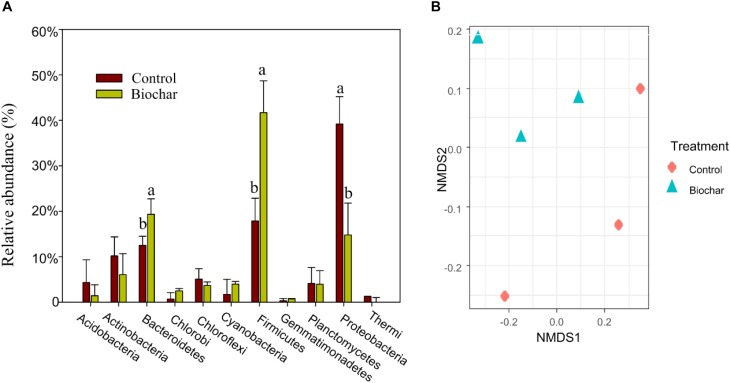
Effect of the biochar amendment on soil bacterial communities in the rhizosphere. **(A)** Taxonomic composition. Significant differences between treatments were tested by one-way ANOVA with Fisher’s *LSD* test, different letters indicate that significance at *P* < 0.05 level. **(B)** Ordination of Bray-Curtis dissimilarities (*k* = 4; stress = 0.04).

In bulk DNA samples, the phyla Firmicutes and Bacteroidetes were the predominant bacterial taxa found in both the biochar and control treatments. However, the biochar application to soil changed the relative abundance of taxa at the phylum level (Figure 3A) as well as their community structure (Figure 3B). Specifically, applying biochar increased (*P* < 0.05) the relative abundances of Firmicutes and Bacteroidetes, whereas the relative abundance of Proteobacteria decreased (*P* < 0.05). At the family level, the biochar treatment increased the relative abundances of *Alicyclobacillaceae*, *Bacillaceae*, *Clostridiaceae*, *Peptococcaceae*, *Ruminococcaceae*, and *Ignavibacteriaceae*, while it significantly decreased the relative abundance of *Oxalobacteraceae* (*P* < 0.05; Supplementary Figure S3).

According to the Chao 1 estimator, the bacterial community of the biochar treatment had significantly higher (*P* < 0.01) species richness compared with the control treatment. However, the observed OTUs (Supplementary Figure S4) and richness estimated by the “breakaway” package (Supplementary Figure S5) were not significantly different between treatments (*P* > 0.05).

### Soil Bacterial Community Compositions Between ^13^CO_2_-Labeled and Unlabeled Fractions

**FIGURE 4:**
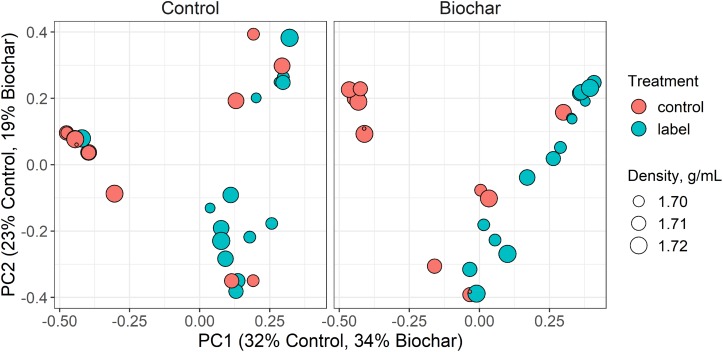
Principal coordinate ordinations (PCoA) ordination based on Bray-Curtis dissimilarities of operational taxonomic units (OTUs) in the heavy (>1.70 g ml^–1^) gradient fractions of the control and biochar treatments. The size of the density legend represents the range of gradient fraction OTU profiles. The size of a point corresponds to its fraction density, while the distance between points represents the similarity in microbial community composition.

Incorporation of ^13^C into microbial DNA was evaluated by investigating the 16S rRNA gene sequence structure between the ^13^C-labeled and unlabeled samples from corresponding density fractions (Figure 4). Sequence results from the ^13^C-labeled samples and their corresponding unlabeled controls diverged specifically in the heavy gradient fraction (Adonis test, *P* < 0.001, Figure 4). This result confirmed the successful incorporation of ^13^C-labeling into the DNA of rhizosphere microbes.

### Bacterial Utilization of Plant-Derived C Stimulated by Biochar Amendment

**FIGURE 5:**
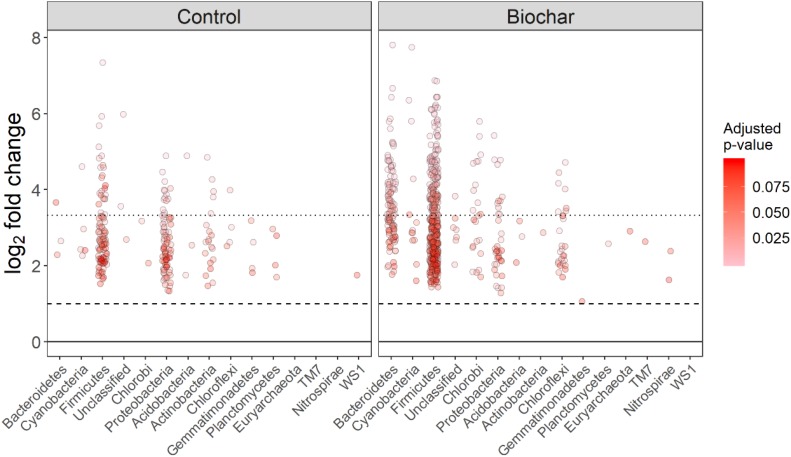
Log_2_-fold changes in the relative abundance of OTUs (^13^C-labeled vs. unlabeled) in the heavy (>1.70 g mL^–1^) gradients for the control and biochar treatments. All the OTUs passed the 35%-sparsity threshold (i.e., OTUs were found in at least 35% fractions) of the heavy fractions. Each circle shows a single OTU, and circles denote proportion fold-changes that had an adjusted *P*-value below a false discovery rate of 10%.

**FIGURE 6:**
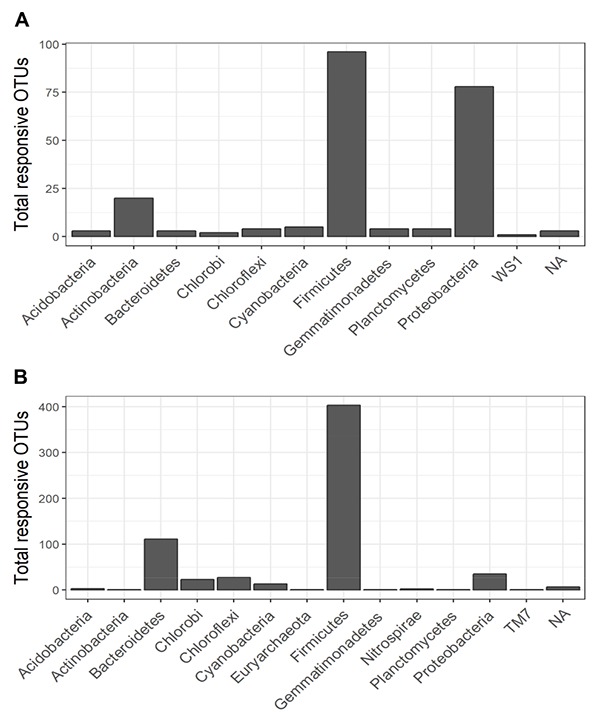
Total responsive OTUs in each bacterial phylum that responded significantly (i.e., with BH-adjusted *P*-values < 0.1) in the heavy gradient fraction of the control **(A)** and biochar **(B)** treatments.

**FIGURE 7:**
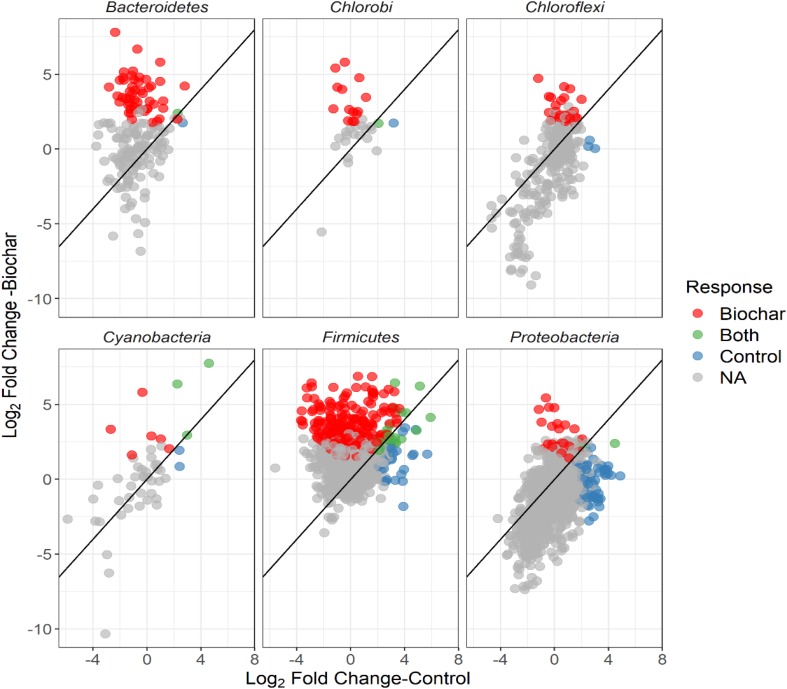
Log_2_-fold changes of the relative abundance of OTUs in response to biochar (^13^C-labeled vs. unlabeled) or control (^13^C-labeled vs. unlabeled) for the top six abundant bacterial phyla. Color of circles indicate unique, shared and non-reposed OTUs between the control and biochar treatments.

The non-normalized OTU abundance matrix taking ^13^C into their DNA was identified by a differential change in their abundance within the heavy gradient fractions when comparing the ^13^C-labeled and unlabeled samples (Figure 5). A total of 6812 and 5589 OTUs passed the 0.35-sparsity threshold filter in the control and biochar treatment, respectively. We observed a wide range of taxa actively utilizing root exudates at the phylum level: namely Firmicutes, Bacteroidetes, Proteobacteria, Chlorobi, and Actinobacteria (Figures 5, 6). In contrast, members of Acidobacteria, Planctomycetes, and Gemmatimonadetes barely used plant-derived C in both control and biochar treatments during 35-day continuous labeling. A total of 223 and 626 labeled OTUs were observed in the control and biochar treatments, which accounted for 3.27 and 11.2% of total OTUs, respectively. Of these labeled bacteria, Firmicutes (96 OTUs) and Proteobacteria (78 OTUs) were the most common taxa utilizing plant-derived C in the control treatment, which accounted for 43 and 35% of total labeled bacteria, respectively. Compared with the control, the biochar application greatly stimulated Firmicutes (403 OTUs) and Bacteroidetes (111 OTUs), while it decreased members of Proteobacteria (35 OTUs) utilizing root exudates; these taxa accounted for 64, 18, and 5.5% of the total labeled bacteria, respectively. The OTUs identified as root-exudate consumers were highly diverse in both treatments (Figure 7). There were 51 shared OTUs with 578 and 172 OTUs were unique to the biochar and control treatments, respectively. The shared OTUs were mainly distributed within the phylum Firmicutes (Figure 7 and Supplementary Figure S6). With regard to the overall taxonomic composition of the bacterial community, the biochar amendment increased the relative abundances of Firmicutes and Bacteroidetes, while decreasing the abundance of Proteobacteria in the > 1.70 g mL^–1^ heavy fractions (Supplementary Figure S7).

**FIGURE 8:**
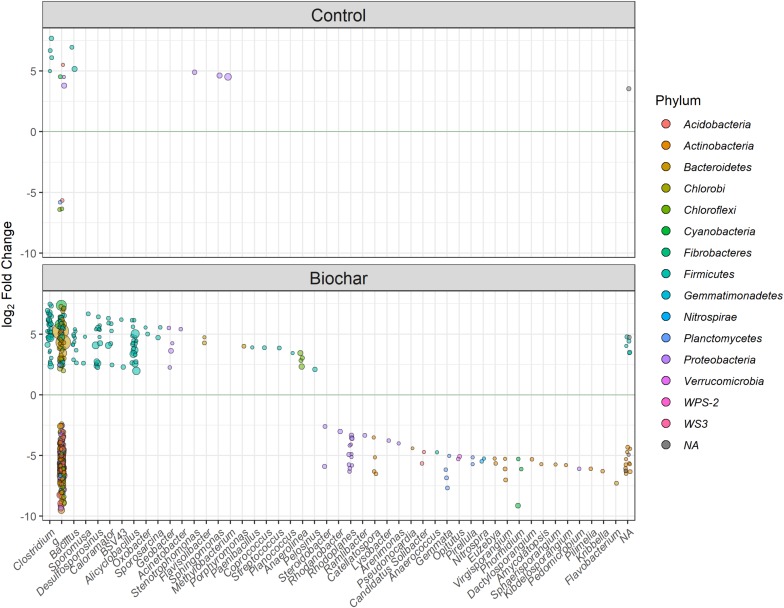
Differentially abundant OTUs between the labeled vs. unlabeled heavy (>1.70 g ml^–1^) gradient fractions for the control and biochar treatments at the genus level. Highly enriched OTUs with a significance of *P* < 0.01 are shown. The g_ indicates unclassified microbe at the genus level; the circle size represents the mean normalized counts of the OTUs across all samples.

At the genus level, we detected a total of 19 genera in the biochar treatment, mainly belonging to *Clostridium*, *Desulfosporosinus*, *Alicyclobacillus*, *Caloramator*, and *Bacillus* (within the phylum Firmicutes), *Flavisolibacter* and *Porphyromonas* (phylum Bacteroidetes), and *Geobacter* (within the phylum Proteobacteria). All of these taxa were significantly enriched (*P* < 0.01) in the heavy fractions of labeled samples relative to the corresponding unlabeled samples (Figure 8). By contrast, in the control treatment, we only detected five genera that were significantly (*P* < 0.01) enriched in its heavy fractions, which belonged to the phyla Firmicutes and Proteobacteria.

## Discussion

Recent studies have shown that using a system of legume-based intercropping can enhance N fixation and phosphorous P availability in the rhizosphere over that of a monoculture (Tang et al., 2014; Rose et al., 2015). In the present study, we found no significant differences in soil available nutrients or plant biomass in the legume-based intercropping system between the biochar and control treatments. The minor differences in soil nutrient availability and plant biomass between treatments can be attributed to multiple factors. For example, the original soil organic matter content in the intercropping system was relatively high, while the biochar rate (2%) used in the study could not high enough to markedly improve soil nutrient conditions or plant grawth. Other, studies have shown that biochar amendment may have the potential to improve soil nutrient status and alleviate soil nutrient stress (Ding et al., 2016; Pandit et al., 2018). Furthermore, Graber et al. (2015) found that substances extracted from biochar have a strong effect on root hair development under abiotic growing conditions.

Phylogenetic affiliation of the 16S rRNA gene sequences revealed that biochar-induced shifts in soil microbial community composition were mainly ascribed to a remarkable increase in the relative abundances of the phyla Firmicutes and Bacteroidetes, as well as a significant decrease in the relative abundance of the phylum Proteobacteria. These results are consistent with those reported by Hu et al. (2014), who found that Firmicutes and Bacteroidetes occurred distinctively in a red-oxidized loam soil after short-term biochar amendment compared with the control samples. Members of Bacteroidetes, widely found in soils and animal-centric canals, have the ability to degrade polysaccharides and cellulose (Fierer et al., 2007; Hu et al., 2014; Wolińska et al., 2017). A study also found that the relative abundance of Bacteroidetes was substantially higher in the root-associated community of biochar-amended soil, while the abundance of Proteobacteria was much higher in the control treatment (Kolton et al., 2011). Firmicutes are recognized as copiotrophs (or r-strategists), which explains their predominance in the soil after biochar amendment. A large proportion of members from the family Clostridiaceae in the phylum Firmicutes are capable of N fixation (Ding et al., 2017). The Chao 1 estimator of the bacterial community greatly increased after biochar amendment, although whereas we did not find significant differences in the observed species. The similarity in observed species between treatments may be attributed to the fact that identifying diversity in complex soil environments is very challenging. Furthermore, the richness estimated by the “breakaway” package did not significantly differ between treatments. The discrepancies between Chao 1 estimator and estimated richness may be attributed to the results of Chao 1 problematic in environments where many rare OTUs are never detected (Willis et al., 2017).

Pulse-labeling plants with ^13^CO_2_ emphasized the current photosynthate, which may not label all plant C pools to the same degree (De Visser et al., 1997; Yao et al., 2012). In the current study, we applied a steady-labeling approach instead of pulse-labeling to ensure that all the plant-derived C was labeled to the same degree. During the 35-day period of continuous labeling with ^13^CO_2_, the biomass of faba bean and maize seedlings was synthesized. At the end of this labeling period, the δ^13^C values of rhizosphere soil were labeled up to 300‰ for both the control and biochar treatments (Supplementary Figure S2). This labeled CO_2_ in soil would have participated in a fair number of processes, including microbial incorporation and the respiration of root exudates. Hence, the experimental set-up used in this study allowed us to explore, in a more direct way, the effects of soil biochar amendment on the interaction between plant and microbes in the rhizosphere. The “cross-feeding” is a common limitation for DNA-SIP approach. Ideally a short sampling time should be selected to reduce the cross-feeding effect, but this is difficult to optimize in practice. If sampling is too late, the rate of label incorporation by primary autotrophic assimilation may be slower than secondary heterotrophic incorporation; if sampling is too early there may be insufficient^13^C label in DNA for accurate detection. Herein the 35-day continuous labeling was performed to generate enough amount of labeled DNA. Despite the above-mentioned limitations and complications, the continuous labeling technique combined with DNA-SIP is the most powerful tool available for identifying the microbial communities primarily utilizing plant-derived C.

For both the unlabeled ^13^CO_2_-labeled and labeled samples, fraction density played a major role in 16S rRNA gene composition. This result was expected because genome G+C content is positively related to DNA buoyant density and therefore influence the 16S rRNA gene sequence structure. Additionally, we observed that the biochar application changed the soil bacterial community composition and diversity in the rhizosphere, while altering the members involved in actively assimilating the plant-derived C. Recent studies have documented that despite the high diversity of microbes occurring in the rhizosphere, only a few microbial groups (less than 4% of the total proportion) effectively utilize the plant-derived C (Gschwendtner et al., 2016; Hannula et al., 2017). In the current study, we found a wide range of bacterial taxa involved in the utilization of plant-derived C, in both control and biochar treatments (Figure 4). These results may suggest that bacterial species from these taxa grew more slowly, and this phenomenon may be attributed to different lifestyle strategies of these taxa. For example, Acidobacteria are recognized as slow-growing bacteria, or “oligotrophs,” which have high C use efficiency and are abundant in soil with high recalcitrant organic matter concentration (Fierer et al., 2007; Trivedi et al., 2017). Similarly, Gemmatimonadetes have been found to decrease their relative abundance after fresh organic matter input, and they are more likely to decompose existing soil organic C rather than fresh organic matter (Pascault et al., 2013; Whitman et al., 2016).

In the control treatment, Firmicutes and Proteobacteria were two predominant bacterial phyla utilizing the plant-derived C. By comparison, the biochar treatment clearly stimulated more bacterial members to utilize the plant-derived C (Figure 4). Specifically, the biochar amendment significantly enhanced the utilization of plant-derived C by Firmicutes and Bacteroidetes members (e.g., *Clostridium*, *Sporomusa*, *Desulfosporosinus*, *Alicyclobacillus*, *Flavisolibacter*, *Geobacter*, and *Bacillus*), however, in the control treatment, Proteobacteria members (e.g., *Methylobacterium* and *Sphingomonas*) tended to increase in their relative abundance to consume the plant-derived *C. Bacillus* and *Clostridium* were found in both the control and biochar treatments, and these two taxa are known, respectively, for their N-fixing and P-solubilizing abilities (Long et al., 2018). Our results imply that *Bacillus* and *Clostridium* may play an important role in N and P cycling in a faba bean-maize intercropping system. Interestingly, *Desulfosporosinus*, *Alicyclobacillus*, and *Geobacter* are known for their iron and sulfur oxidation abilities (Ding et al., 2015). Therefore, the participation of these taxa in nutrient cycling (e.g., by fixing N or solubilizing P) should be addressed in the intercropping system in future research. Generally, the pronounced shifts in the soil bacterial community utilizing the plant-derived C provide strong evidence that biochar amendment induces a distinct plant-microbiome interaction in the rhizosphere.

Our results indicate that biochar amendment not only changed the overall bacterial community in the rhizosphere but also favored the presence and abundance of bacteria with N-fixing and P-solubilizing abilities in the legume-based intercropping system (*V. faba* L., with *Z. mays* L.). Their utilization of plant-derived C may thus have crucial consequences for C storage and dynamics, as well as nutrient cycling and plant performance more generally, in agro-ecosystems that use intercropping.

## Author Contributions

HY designed the study. YL and HL performed the experiments. YL and HY analyzed the data. HL and HY wrote the manuscript.

## Conflict of Interest Statement

The authors declare that the research was conducted in the absence of any commercial or financial relationships that could be construed as a potential conflict of interest.
