# Supplementary material for: Biochar Amendment Stimulates Utilization of Plant-Derived Carbon by Soil Bacteria in an Intercropping System
Source: Front Microbiol. 2019 Jun 18;10:1361. doi: 10.3389/fmicb.2019.01361 (PMC6611431; doi:10.3389/fmicb.2019.01361)
Supplement: Supplementary file 1 [file Data_Sheet_1.docx]

*Supplementary material*

Biochar amendment stimulates utilization of plant-derived carbon by soil bacteria in an intercropping system

**Hongkai Liao^1,2,3,4^ ·Yaying Li^1,2,*^· Huaiying Yao^1,2,5,*^**

^1^ Key Laboratory of Urban Environment and Health, Institute of Urban Environment, Chinese Academy of Sciences, Xiamen 361021, People’s Republic of China

^2^ Key Laboratory of Urban Environmental Processes and Pollution Control, Ningbo Urban Environment Observation and Research Station-NUEORS, Chinese Academy of Sciences, Ningbo 315800, People’s Republic of China

^3^ University of Chinese Academy of Sciences, Beijing 100049, People’s Republic of China

^4^ Guizhou Provincial Key Laboratory of Mountain Environment, Guizhou Normal University, Guiyang 550001, People’s Republic of China

^5^ Research Center for Environmental Ecology and Engineering, School of Environmental Ecology and Biological Engineering, Wuhan Institute of Technology, Wuhan 430073, People’s Republic of China

For correspondence. * E-mail: hyyao@iue.ac.cn; yyli@iue.ac.cn. Tel: +86-0592-6190791; Fax: +86-0592-6190791


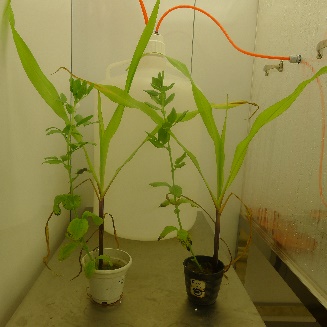


**Figure S1** Plants in the pots after 35-day continuous of labeling


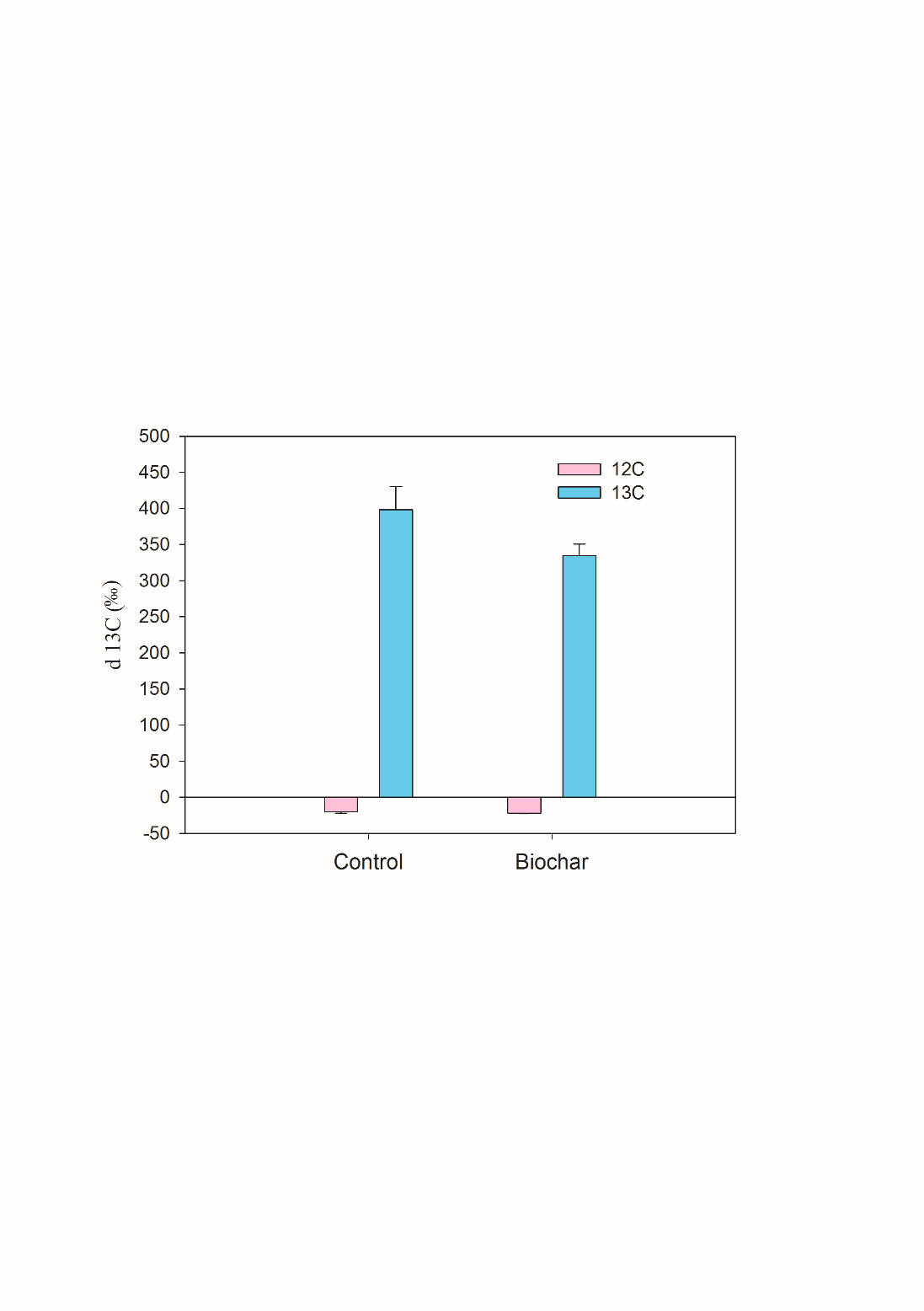


**Figure S2** Incorporation of ^13^C-labelled rhizodeposits into the soils after 35-days of continuous labeling. Bars present means ± SE.


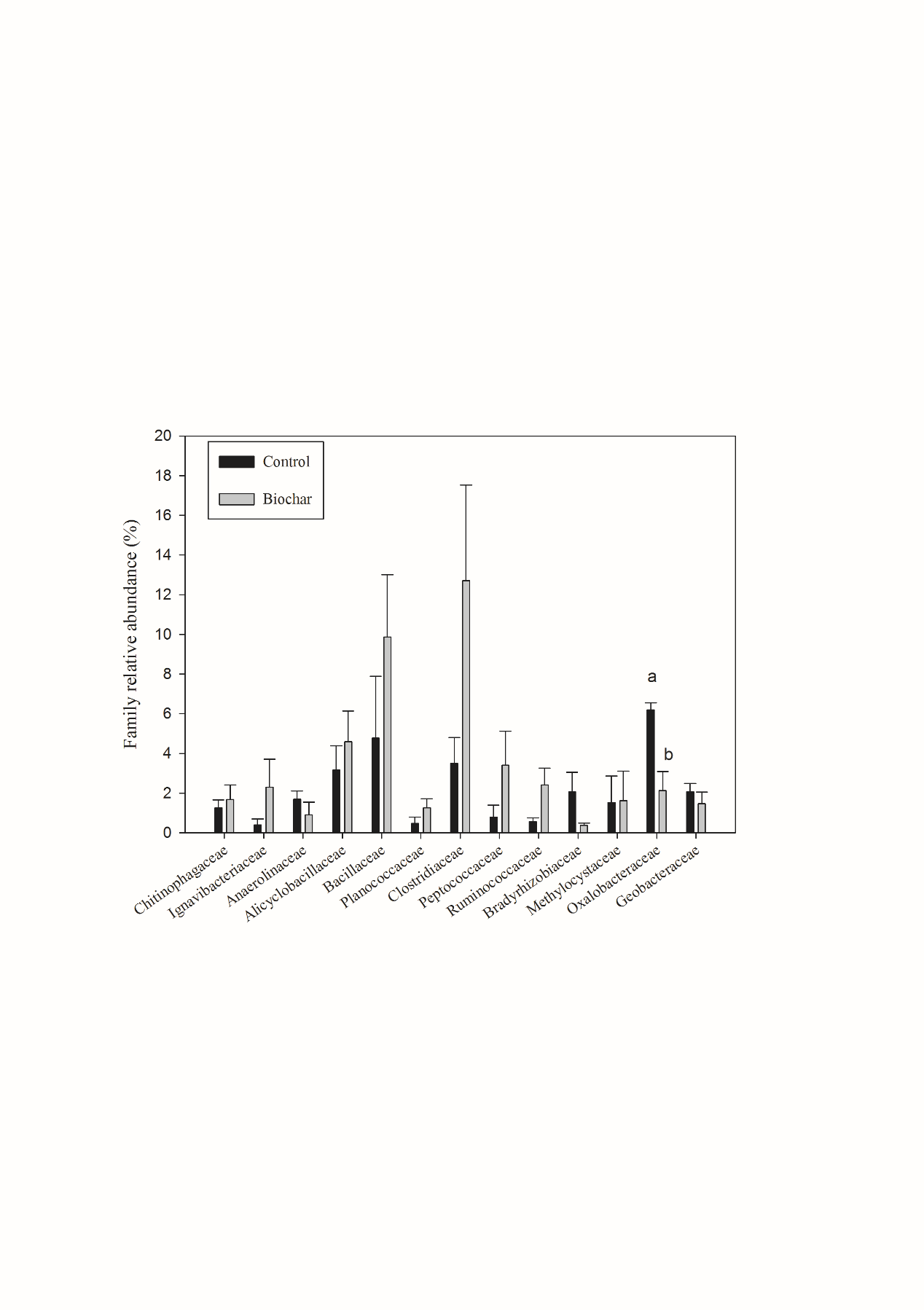


**Figure S3** The relative abundance of rhizosphere bacterial families determined by high-throughput sequencing of the 16S rRNA gene amplicons. Statistically significant differences between group pairs were determined by the *LSD* at *P* < 0.05 level. Bars present means ± SE.


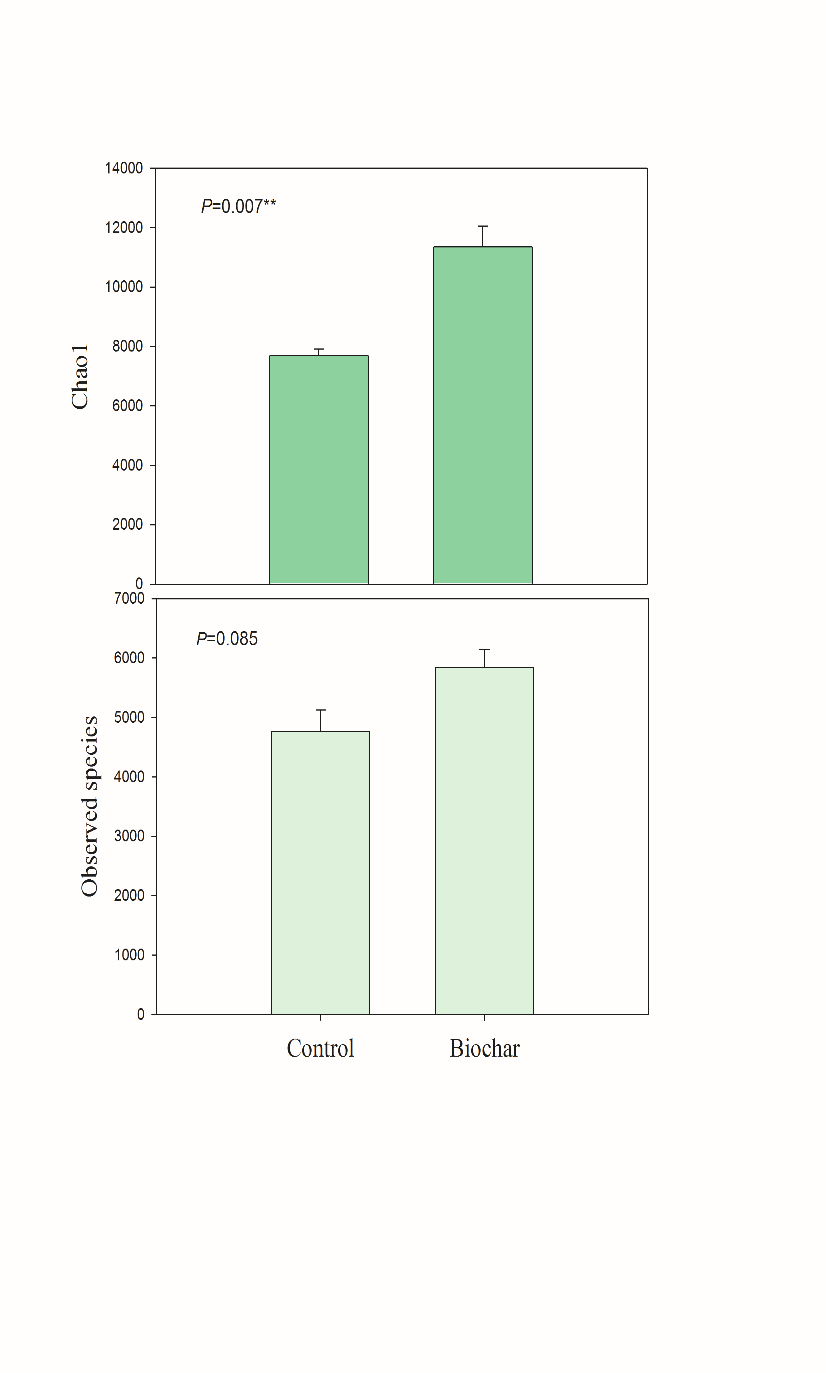


**Figure S4** Effect of the biochar amendment on the alpha diversity of the soil microbial communities. Bars are means ± SE.


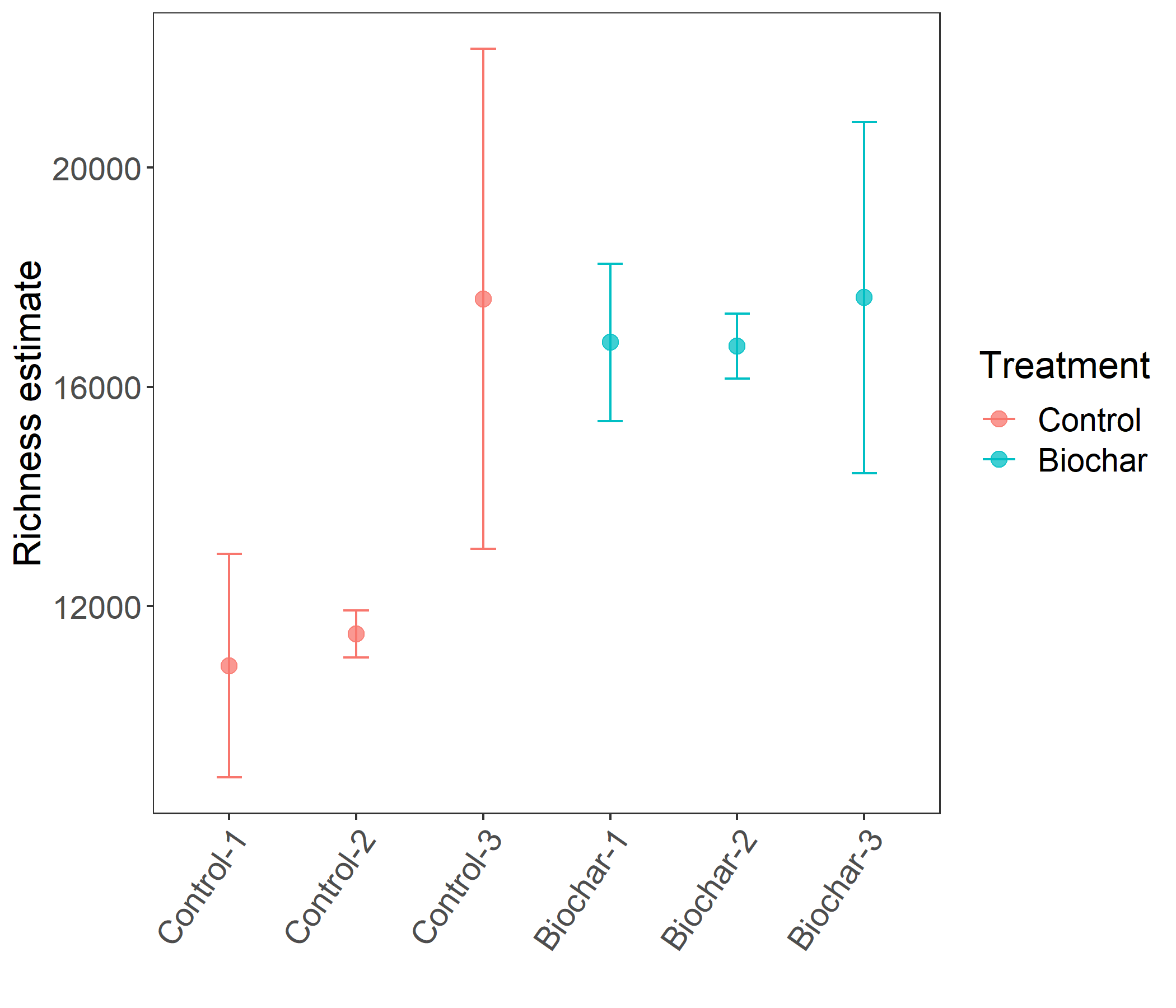


**Figure S5** Effect of the biochar amendment on the bacterial richness as estimated by ‘breakaway’ package. Bars are means ± SE.


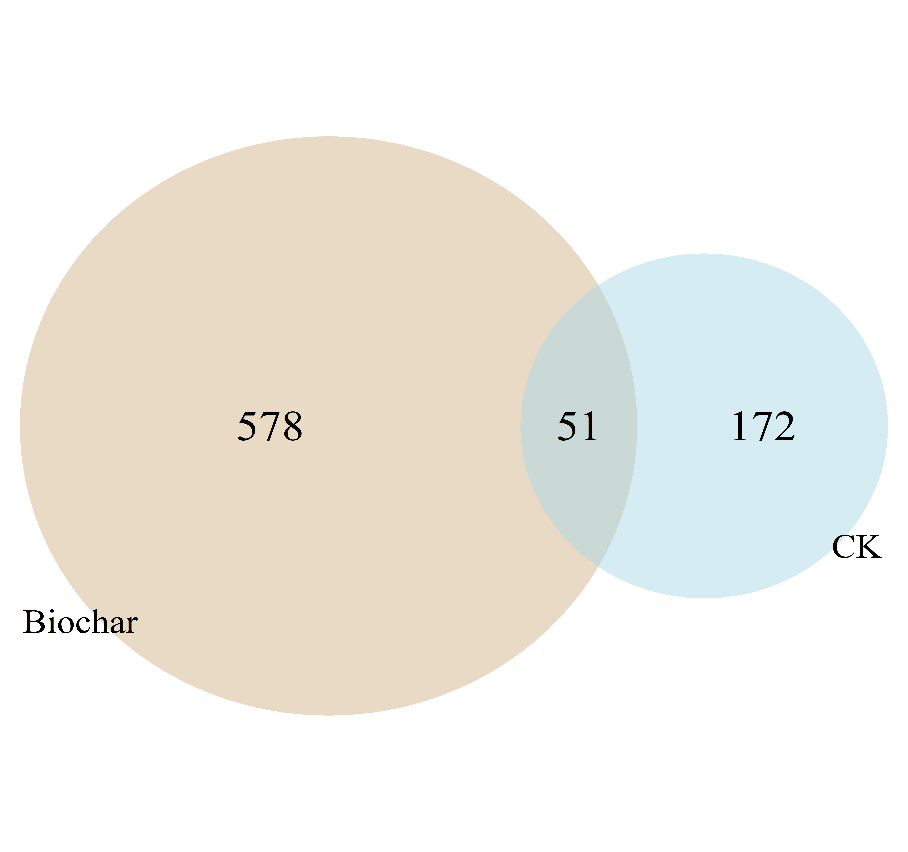


**Figure S6** Overlap of responsive OTUs between biochar and control treatments

**
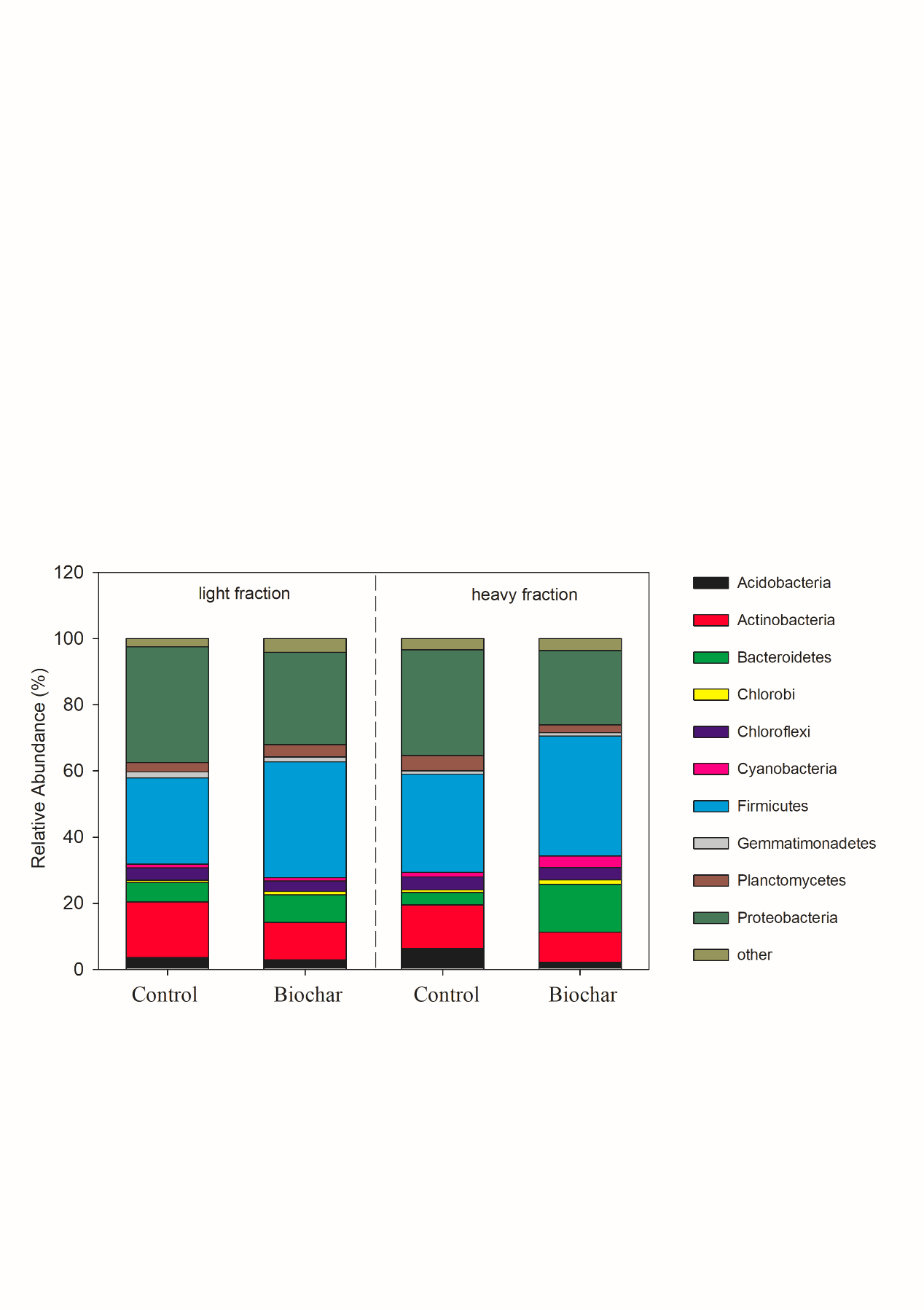
**

**Figure S7** Taxonomy of the microbial communities in the light (<1.69 g ml^-1^) and heavy (>1.70 g ml^-1^) gradient fractions of the control and biochar treatments.
